# Characterization of Alternative Promoters to Stagger and Control Protein Expression in the Baculovirus-Insect Cell System: From Intracellular Reporter Proteins to Fluorescent Influenza Virus-like Particles

**DOI:** 10.1186/1753-6561-9-S9-P49

**Published:** 2015-12-14

**Authors:** Steve George, Marc G Aucoin

**Affiliations:** 1 Department of Chemical Engineering, University of Waterloo, Waterloo, Ontario, N2L3G1, Canada

## Background

The Baculovirus Expression Vector System (BEVS) is increasingly used for protein production in both industry and academia, and much work has been conducted to improve this system. The baculovirus infection of an insect cell sets up a sophisticated and complex series of gene expression events that are very tightly temporally regulated. The study of this system has progressed to such an extent that many control elements, such as activators, enhancers, and promoters involved in this process have been discovered and characterized to some extent, as reviewed in [1]. These control elements can be used to regulate the expression of heterologous genes, in order to move beyond "brute force" expression of large amounts of protein within insect cells. It enables researchers to set up a pre-planned series of expression events of multiple genes within one cell, and to essentially "program" gene expression by modifying the baculovirus genome. While some groups have investigated this, a systematic study of control elements and how expression from a single gene affects expression from other heterologous genes, has not been conducted thus far. This study characterizes gene expression from several baculovirus promoters for the production of proteins and virus-like particles, and examines interaction effects when promoters drive expression of genes at different times and at different levels.

## Materials and Methods

Two sets of protein coding genes were investigated. Both sets of constructs were arranged such that one gene was always under the control of the very strong polyhedron (polh) promoter, while the other gene was under the control of the early ie1, late basic, gp64orvcath, or the very late p10 promoters. The first set of proteins examined consisted of two easily traceable fluorescent proteins requiring minimal post-translational processing: the enhanced green fluorescent protein (eGFP, herein referred to as GFP) and a red fluorescent protein (DsRed2 herein referred to as RFP). The RFP gene was always under the control of the polh promoter while GFP was placed downstream of one of the other five promoters [2].The second set of proteins studied were fusions of influenza A virus proteins. More specifically, human influenza A/PR/8/34 hemagglutinin (HA) and matrix (M1) proteins were fused to eGFP(HAGFP) and DsRed2 (M1RFP) respectively. The M1RFP gene was always under the control of the polh promoter while HAGFP was placed downstream of one of the other five promoters.

Sf9 cells were infected at a cell density of 1x106 cells/mL and at a multiplicity of infection of 5. Cells were examined by light and fluorescence microscopy, as well as by flow cytometry. Virus-like particles were recovered from infected cell culture supernatants by subjecting the supernatants to iodixanol gradient ultracentrifugation as previously described in [3]. Virus-like particles were characterized by flow cytometry using a method similar to that described in [4],by negative stain transmission electron microscopy (TEM) and by multi-angle dynamic light scattering (MADLS).

## Results

By keeping the RFP gene under the control of the polh promoter and varying the promoter in front of the GFP gene, the effect of the expression of one protein on another was established. As hypothesized by us and alluded to by others, high expression levels of one protein (GFP) results in lower levels of the other (RFP), with the exception of GFP driven by the ie1 promoter, which resulted in lower than anticipated levels of RFP. These findings are now allowing the development of mathematical models with promoter specific parameters.

**Figure 1:**
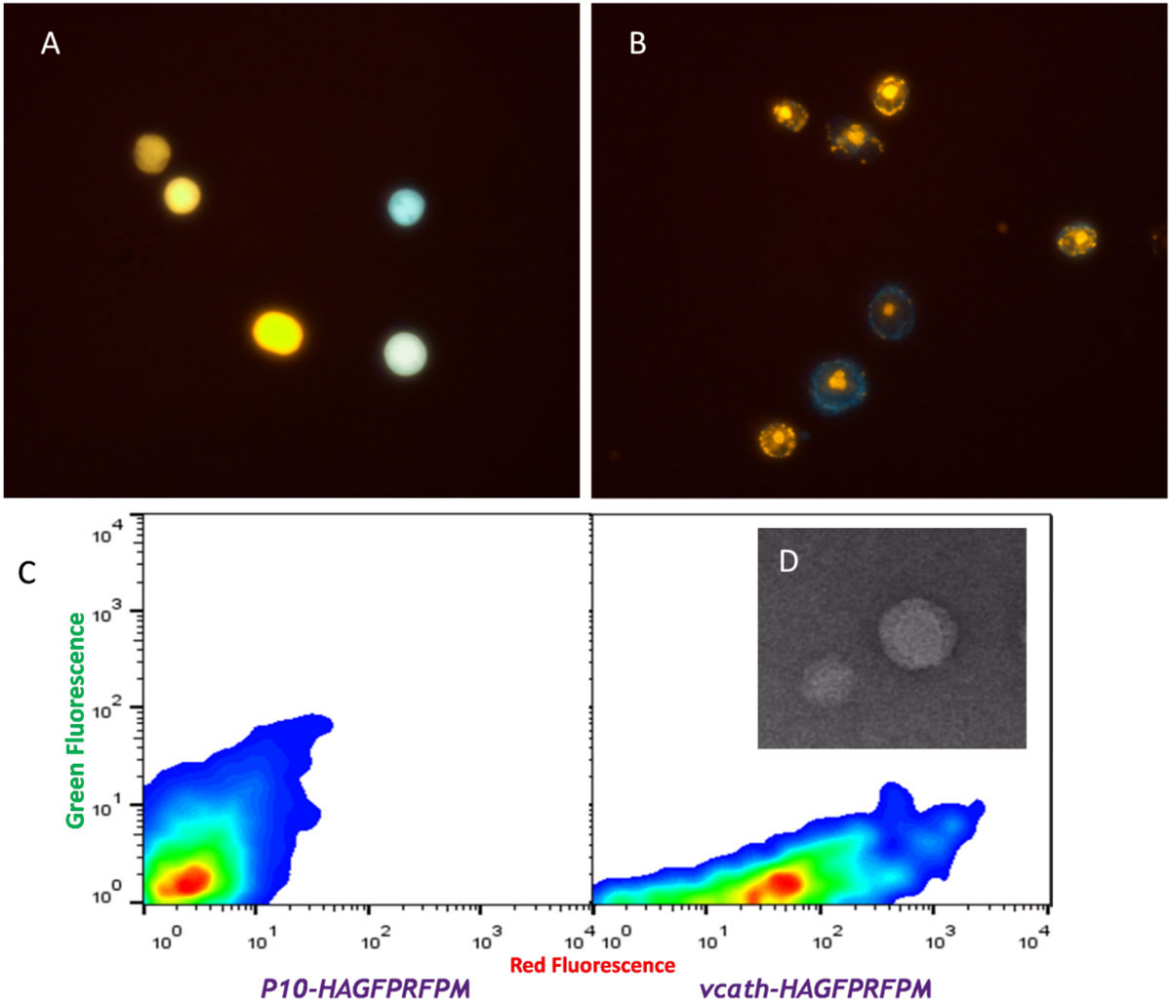
**(a) Uniform fluorescence in cells infected with baculovirus carrying GFP and RFP under two different promoters**. (b) Localized protein expression in cells infected with baculovirus carrying HAGFP and M1RFP under two different promoters. (c) Smoothed flow cytometry scatterplots of iodixanol gradient purified VLP samples. Left scatterplot is the analysis of material recovered from the supernatant of insect cell cultures infected with a baculovirus having the *HAGFP *gene under the control of the *p10 *promoter and *M1RFP *under the control of the *polh *promoter. Right scatterplot is the analysis of material recovered from the supernatant of insect cell cultures infected with a baculovirus having the *HAGFP *gene under the control of the *vcath *promoter and *M1RFP *under the control of the *polh *promoter. (d) TEM image of VLPs produced from cells infected with a baculovirus having the *HAGFP *gene under the control of the *p10 *promoter and *M1RFP *under the control of the *polh *promoter.

Compared to the production of GFP and RFP driven by various baculovirus promoters upon infection of insect cells, levels of fluorescence in cells infected with baculovirus carrying the fluorescent influenza protein fusion genes, under the control of the same promoter combinations, were very similar. This was true despite the fusion proteins having completely different localization in the cell. While GFP and RFP 'flooded' the cell to create cells with uniform fluorescence (Figure 1A), the fusion proteins had distinct localization. HAGFP, localized uniquely to the cell membrane while M1RFP first localized near the center of the cell before also localizing at the cell membrane (Figure 1B).

Unlike the original model system consisting of GFP and RFP, the goal of making the influenza protein fusion constructs was the development of a system that would lead to fluorescent virus-like particles. Given the very similar fluorescent time-course profiles of the cells, there were no immediate indicators of VLP budding. Iodixanol gradient ultracentrifugation of the supernatants was able to isolate VLPs of approximately 70-85 nm having the distinct spike-like projections of influenza particles (Figure 1D). Iodixanol purified VLPs subjected to analysis by flow cytometry revealed different red and green fluorescent levels corresponding to the choice of baculovirus vector and promoter control used for the generation of the particles (Figure 1C).

## Conclusions

This work shows that promoter control is achievable in a system producing simple proteins, as well as in one producing complex protein structures such as virus-like particles. Characterization of outcomes from the use of promoter control is leading to more robust, fine-tunable and predictable protein expression. To our knowledge, this work is the first instance where the complete HA protein has been fused to a fluorescent protein, and the first report of fluorescently tagged HA and M1 proteins co-localizing into a single particle, which can be tracked by flow cytometry. Finally, it has been shown that tailored protein expression can also modulate the composition of virus-like particles.

## Acknowledgements

The authors would like to thank the following individuals and organizations: Dr. Maud Gorbet, Altamash Jauhar, Sascha Keißlich, Jennifer Mackenzie, Emma Dare and the Natural Sciences and Engineering Research Council of Canada for supporting this work.
